# Examining Social Determinants of Health During a Pandemic: Clinical Application of Z Codes Before and During COVID-19

**DOI:** 10.3389/fpubh.2022.888459

**Published:** 2022-04-29

**Authors:** Xueying Yang, Brooks Yelton, Shujie Chen, Jiajia Zhang, Bankole A. Olatosi, Shan Qiao, Xiaoming Li, Daniela B. Friedman

**Affiliations:** ^1^Center for Healthcare Quality, Arnold School of Public Health, University of South Carolina, Columbia, SC, United States; ^2^Department of Health Promotion, Education, and Behavior, Arnold School of Public Health, University of South Carolina, Columbia, SC, United States; ^3^Department of Epidemiology and Biostatistics, Arnold School of Public Health, University of South Carolina, Columbia, SC, United States; ^4^Department of Health Services Policy and Management, Arnold School of Public Health, University of South Carolina, Columbia, SC, United States; ^5^Office for the Study of Aging, Department of Health Promotion, Education, and Behavior, Arnold School of Public Health, University of South Carolina, Columbia, SC, United States

**Keywords:** healthy people 2030, Z codes, social determinants of health, coronavirus, COVID-19, population health

## Abstract

Recognition of the impact of social determinants of health (SDoH) on healthcare outcomes, healthcare service utilization, and population health has prompted a global shift in focus to patient social needs and lived experiences in assessment and treatment. The International Classification of Diseases, 10th Revision, Clinical Modification (ICD-10-CM) provides a list of non-billable “Z codes” specific to SDoH for use in electronic health records. Using population-level analysis, this study aims to examine clinical application of Z codes in South Carolina before and during the COVID-19 pandemic. The study population consists of South Carolina residents who had a healthcare visit and had their COVID-19 test result reported to the state's Department of Health and Environmental Control before January 14, 2021. Of the 1,190,531 individuals in the overall sample, Z codes were used only for 14,665 (1.23%) of the patients, including 2,536 (0.97%) COVID-positive patients and 12,129 (1.30%) COVID-negative patients. Compared with hospitals that did not use Z codes, those that did were significantly more likely to have higher bed capacity (*p* = 0.017) and to be teaching hospitals (*p* = 0.03), although this was significant only among COVID-19 positive individuals. Those at inpatient visits were most likely to receive Z codes (OR: 5.26; 95% CI: 5.14, 5.38; *p* < 0.0001) compared to those at outpatient visits (OR: 0.07; 95%CI: 0.06, 0.07; *p* < 0.0001). There was a slight increase of Z code use from 2019 to 2020 (OR: 1.33, 95% CI: 1.30, 1.36; *p* < 0.0001), which was still significant when stratified by facility type across time. As one of the first studies to examine Z code use among a large patient population, findings clearly indicate underutilization by providers. Additional study is needed to understand the potentially long-lasting health effects related to SDoH among underserved populations.

## Introduction

Healthy People 2030 has increasingly focused on social determinants of health (SDoH) to improve health equity (1). It defines SDoH as “the conditions in the environments where people are born, live, learn, work, play, worship, and age that affect a wide range of health, functioning, and quality-of-life outcomes and risks” (2). Recognition of the impact of SDoH on healthcare outcomes and healthcare service utilization has prompted a global shift in focus away from simply addressing symptoms and conditions to considering patient social needs and lived experiences in assessment and treatment. While conceptualization of SDoH across sectors and entities varies widely, and different approaches are needed to tackle upstream and downstream factors, attention to SDoH is necessary to improve population health (1, 3, 4).

The coronavirus disease 2019 (COVID-19) pandemic, which is caused by the severe acute respiratory syndrome coronavirus 2 (SARS-CoV-2), has exacerbated adverse social conditions, including food and housing insecurity, unemployment, and social isolation, resulting in increased reports of medical issues. With far-reaching, multilayer impacts on SDoH, the COVID-19 pandemic presents a critical time for provider consideration of patient social needs to address consequential health and mental health outcomes resulting from racial and ethnic disparities. Persistent disparities exist for comorbid health conditions, for which SDoH are known drivers, and disproportionate COVID-19 hospitalizations and deaths among minority populations largely point to a variety of overlapping social risk factors (5–7). In addition, many pandemic-related impacts increased the risk profile for certain individuals, particularly those not identifying as White or those identifying as multiracial (8). For example, occupational segregation in the US places a greater proportion of already historically marginalized populations on the “front lines,” and often in positions lacking adequate employee benefits such as health insurance and paid leave (9, 10). Economic losses and school closures also threatened food and housing security for many, creating unfavorable conditions for both chronic disease management and prevention of COVID-19 (10). Increased instances of domestic or intimate partner violence also arose during the pandemic (11, 12), and a variety of population segments experienced increased isolation and loneliness (13). Taken together, SDoH both increase the impact of COVID-19 and contribute to overall morbidity and mortality as the pandemic continues to generate social, economic, and physical losses, while simultaneously complicating healthcare access and quality (14, 15). While the long-term health and mental health impacts are yet to be understood, healthcare providers can begin to document and consider SDoH in assessment and the development of treatment plans to stem preventable adverse health outcomes associated with patient social needs.

The International Classification of Diseases, 10th Revision, Clinical Modification (ICD-10-CM) provides a list of non-billable codes specific to SDoH classified under “Z codes” (Z55–Z65) for use in patient electronic health records (16). There has been an emphasis on increasing the use of underutilized Z codes which are critical to provider understanding of the social and environmental factors impacting patient and community health. However, until guidelines were updated in 2019, obscurity regarding who could utilize these codes led to infrequent application in healthcare settings. In addition, the overwhelming volume of medical codes, the potential lack of provider knowledge of SDoH, limited time with patients, and confusion around appropriate referrals serve to further inhibit standard use (17, 18). Given these barriers, health service administrators and support organizations, such as the American Hospital Association, have worked to promote the use of Z codes to improve individual patient health outcomes and guide intervention at the community and population levels (19). There is great potential for collaboration across sectors to support healthcare in effective utilization of Z code documentation for improved patient health. For example, academic-clinical-community partnerships in South Carolina are working to improve health equity by addressing SDoH at multiple system levels (20, 21).

The purpose of this study was to examine clinical application of Z codes in South Carolina both before and during the COVID-19 pandemic (using March 6, 2020, the date of the first COVID-19 diagnosis in South Carolina, as cutoff). Specifically, we aimed to (1) describe the frequency of Z code use among a statewide patient population in South Carolina and examine demographic variations (e.g., age group, race and ethnicity, urban/rural residence) in this coding; (2) evaluate Z code use by facility characteristics (e.g., facility bed size, facility type), type of patient visit (e.g., in-patient, outpatient, emergency room) and medical specialty type; and (3) examine whether Z code use differed by healthcare visit and medical specialty according to patient race and ethnicity.

## Methods

### Population and Data Sources

The study population consists of South Carolina residents that had at least one healthcare visit between January 2, 2019 and March 6, 2020 (i.e., before COVID-19 pandemic), and a subsequent healthcare visit and COVID-19 test result (both positive and negative) reported to the South Carolina Department of Health and Environmental Control (SC DHEC) between March 7, 2020 and January 14, 2021. The sources of data include clinician reports, laboratory reports, reports by other entities (e.g., hospitals, poison centers), death certificates, and hospital discharge or outpatient records. The criteria of case ascertainment of COVID-19 were described in the standardized surveillance case definition of COVID-19 (22). That is, the information from a statewide Case Report Form (CRF) (“Human Infection with 2019 Novel Coronavirus Case Report Form”) for SARS-CoV-2 infection was used to define COVID-19 positive populations (including both confirmed and probable cases) (23). A total of 1,190,531 individuals who were either COVID-19 positive (*n* = 260,344) or COVID-19 negative (*n* = 930,187) were included in the current study. Human subjects approval was obtained from the institutional review boards at the University of South Carolina and relevant SC state agencies.

### Variables

The primary variable of interest was the coding of a Z code indicative of a SDoH, specifically ICD-10 codes Z55 through Z65. These codes are used to characterize socioeconomic and psychosocial situations, such as problems related to education and literacy, employment, occupational exposure, and housing, that can impact health and mental health (19). To capture SDoH use in different types of medical encounters with healthcare systems, we categorized the healthcare visits by facility type and physician specialty. For facility type, we classified healthcare visits as visiting emergency room (ER), inpatient service (IP), and outpatient service (OP). For healthcare visits by physician specialties, we selected areas with the most frequent medical encounters (e.g., numbers of visits), such as emergency medicine (EM), internal medicine (IM), and gastroenterology, or with the largest proportions of patients receiving any Z codes (e.g., addiction psychiatry [AP]).

### Data Analysis

Descriptive statistics, including frequencies and percentages, were used to examine categorical variables. The variable distributions of individual demographics, hospital characteristics, and healthcare visits with and without SDoH coding were summarized and compared using Chi-square tests. Logistic regression models were used to estimate the associations between SDoH coding and socio-demographic characteristics. The use of SDoH coding before and during COVID-19 was also examined by demographics using logistic regressions. Using bar charts, we also described the SDoH coding among racial and ethnic groups, stratified by time (i.e., before COVID-19: 1/2/2019-3/5/2020; during COVID-19: 3/6/2020-1/14/2021) and by medical specialty visits. Significance was set at *p* < *0.05*.

## Results

### Most Commonly Utilized Z Codes

Of the 1,190,531 individuals in the overall sample, Z codes were used only for 1.23% of the patient population (14,665 patients), including 0.97% (2,536) COVID-positive patients and 1.30% (12,129) COVID-negative patients. Applied Z codes fell within nine categories of codes. The categories in which most Z codes were used included: *Problems related to housing and economic circumstances* (Z59; *n* = 6,296), *Problems related to upbringing* (Z62; *n* = 3,845), and *Other problems related to primary support group, including family circumstances* (Z63; *n* = 2,905). Z codes were significantly used more often among those who tested negative for COVID-19 in Z56 (Problems related to employment and unemployment), Z59 (Problems related to housing and economic circumstances), and Z60 (Problems related to social environment); while less often for Z57 (Occupational exposure to risk factors), Z63.4 (Disappearance and death of family member), Z64.1 (Problems related to multiparity), and Z65 (problems related to other psychosocial circumstances). Table 1 presents the frequencies and percentages of most commonly used Z code categories and sub-categories among overall sample and by COVID-19 status.

**Table 1:** Most commonly used Z codes: Overall and by covid-19 status.

**Z code**	**Overall (n, %)**	**COVID- (n, %)**	**COVID+ (n, %)**	***P-value***
**Z55 (Problems related to education and literacy)**	103 (0.009)	83 (0.009)	20 (0.008)	0.5672
Z55.9 (Problems related to education and literacy, unspecified)	40 (0.003)	–	–	0.3832
Z55.8 (Other problems related to education and literacy)	26 (0.002)	–	–	0.7937
Z55.4 (Educational maladjustment and discord with teachers and classmates)	17 (0.001)	–	–	0.5465
**Z56 (Problems related to employment and unemployment)**	1,785 (0.15)	1,533 (0.165)	252 (0.097)	**0.0002**
Z56.0 (Unemployment, unspecified)	1,658 (0.139)	1,427 (0.153)	231 (0.089)	**0.0001**
Z56.3 (Stressful work schedule)	42 (0.004)	–	–	0.4779
Z56.6 (Uncongenial work environment)	37 (0.003)	–	–	0.5428
**Z57 (Occupational exposure to risk factors)**	140 (0.012)	106 (0.011)	34 (0.013)	**0.0279**
Z57.5 (Occupational exposure to toxic agents in other industries)	35 (0.003)	–	–	0.3835
Z57.8 (Occupational exposure to other risk factors)	29 (0.002)	16 (0.002)	13 (0.005)	**<.0001**
Z57.4 (Occupational exposure to toxic agents in agriculture)	20 (0.002)	–	–	0.7487
**Z59 (Problems related to housing and economic circumstances)**	6,296 (0.529)	5,402 (0.581)	894 (0.343)	**<.0001**
Z59.0 (Homelessness)	4,848 (0.407)	4,190 (0.45)	658 (0.253)	**<.0001**
Z59.9 (Problem related to housing and economic circumstances, unspecified)	1,002 (0.084)	864 (0.093)	138 (0.053)	**0.0023**
Z59.6 (Low income)	383 (0.032)	327 (0.035)	56 (0.022)	0.1613
**Z60 (Problems related to social environment)**	1,246 (0.105)	997 (0.107)	249 (0.096)	**0.0086**
Z60.2 (Problems related to living alone)	668 (0.056)	519 (0.056)	149 (0.057)	**0.0005**
Z60.8 (Other problems related to social environment)	283 (0.024)	234 (0.025)	49 (0.019)	0.9923
Z60.9 (Problem related to social environment, unspecified)	226 (0.019)	183 (0.02)	43 (0.017)	0.4874
**Z62 (Problems related to upbringing)**	3,845 (0.323)	3,186 (0.343)	659 (0.253)	0.7692
Z62.810 (Personal history of physical and sexual abuse in childhood)	3,050 (0.256)	2,529 (0.272)	521 (0.2)	0.7293
Z62.820 (Parent-biological child conflict)	507 (0.043)	413 (0.044)	94 (0.036)	0.4497
Z62.21 (Parental overprotection)	311 (0.026)	243 (0.026)	68 (0.026)	**0.0312**
**Z63 (Other problems related to primary support group, including family circumstances)**	2,905 (0.244)	2,390 (0.257)	515 (0.198)	0.4886
Z63.8 (Other specified problems related to primary support group)	944 (0.079)	792 (0.085)	152 (0.058)	0.3171
Z63.4 (Disappearance and death of family member)	859 (0.072)	661 (0.071)	198 (0.076)	**<.0001**
Z63.0 (Problems in relationship with spouse or partner)	432 (0.036)	364 (0.039)	68 (0.026)	0.3866
**Z64 (Problems related to certain psychosocial circumstances)**	80 (0.007)	62 (0.007)	18 (0.007)	0.2169
Z64.0 (Problems related to unwanted pregnancy)	–	–	–	0.9676
Z64.1 (Problems related to multiparity)	43 (0.004)	30 (0.003)	13 (0.005)	**0.0246**
Z64.4 (Discord with counselors)	31 (0.003)	–	–	0.5177
**Z65 (Problems related to other psychosocial circumstances)**	1,099 (0.092)	801 (0.086)	298 (0.114)	**<.0001**
Z65.1 (Imprisonment and other incarceration)	598 (0.05)	389 (0.042)	209 (0.08)	**<.0001**
Z65.3 (Problems related to other legal circumstances)	197 (0.017)	174 (0.019)	23 (0.009)	**0.0358**
Z65.8 (Other specified problems related to psychosocial circumstances)	167 (0.014)	137 (0.015)	30 (0.012)	0.8176
Z65.9 (Problem related to unspecified psychosocial circumstances)	119 (0.01)	86 (0.009)	33 (0.013)	**0.0025**

*Note: Per SC DHEC data use policy, categories with cell size less than 10 are not reported to protect patient privacy. Values demonstrating statistical significance are bolded*.

### Z Codes by Demographics

As shown in Table 2, older individuals and those identified as Black or Hispanic within the sample had significantly lower odds of receiving at least one Z code; however, those identified as male or urban resident had higher odds of receiving at least one Z code. These findings are similar when examining COVID-19 positive and negative populations separately, but lower odds of a Z code amongst Hispanic or Latino residents was not found to be significant (*p* = 0.11–0.23). As indicated in Table 3, examining use of Z codes across time revealed a higher rate of Z codes use during COVID-19 than before the pandemic among nearly all demographic groups.

**Table 2:** Demographic characteristics of individuals with Z codes (N = 1,190,531).

**Demographic characteristics**	**Overall sample *N* = 1,190,531 N (%)**	**No Z code *N* = 1,175,866 N (%)**	**Z code *N* = 14,665 N (%)**	Adjusted odds ratio/OR^*^(95% CI)	***P-value***
**Age group**					
<18	148,495 (12.47)	146,190 (12.43)	2,305 (15.72)	**1.17 (1.11,1.24)**	**<0.0001**
18-29	177,570 (14.92)	174,886 (14.87)	2,684 (18.3)	Ref	
30-39	151,716 (12.74)	149,312 (12.7)	2,404 (16.39)	**0.87 (0.82,0.92)**	**<0.0001**
40-49	141,167 (11.86)	139,042 (11.82)	2,125 (14.49)	**0.64 (0.60,0.68)**	**<0.0001**
50-59	179,316 (15.06)	176,997 (15.05)	2,319 (15.81)	**0.45 (0.42,0.47)**	**<0.0001**
60+	392,267 (32.95)	389,439 (33.12)	2,828 (19.28)	**0.19 (0.18,0.20)**	**<0.0001**
**Gender**					
Female	655,406 (55.05)	648,487 (55.15)	6,919 (47.18)	Ref	
Male	474,541 (39.86)	467,359 (39.75)	7,182 (48.97)	**1.52 (1.47,1.57)**	**<0.0001**
Unknown/Missing	60,584 (5.09)	60,020 (5.10)	564 (3.85)	1.00 (0.92,1.09)	0.9865
**Race**					
White	471,798 (39.63)	465,501 (39.59)	6,297 (42.94)	Ref	
Black	256,694 (21.56)	253,072 (21.52)	3,622 (24.7)	**0.90 (0.86,0.94)**	**<0.0001**
Asian	4,213 (0.35)	4,190 (0.36)	23 (0.16)	**0.41 (0.27,0.62)**	**<0.0001**
Other/Unknown	457,826 (38.46)	453,103 (38.53)	4,723 (32.21)	**0.74 (0.71,0.77)**	**<0.0001**
**Ethnicity**					
Not Hispanic or Latino	543,695 (45.67)	536837 (45.65)	6,858 (46.76)	Ref	
Hispanic or Latino	29,843 (2.51)	29,530 (2.51)	313 (2.13)	**0.87 (0.77,0.97)**	**0.0157**
Unknown/Missing	616,993 (51.83)	609,499 (51.83)	7,494 (51.1)	**1.12 (1.08,1.17)**	**<0.0001**
**Residence**					
Rural	173,446 (14.57)	171,686 (14.6)	1,760 (12)	Ref	
Urban	1,002,398 (84.2)	989,716 (84.17)	12,682 (86.48)	**1.26 (1.20,1.33)**	**<0.0001**
Missing	14,687 (1.23)	14,464 (1.23)	223 (1.52)	**1.55 (1.35,1.79)**	**<0.0001**

* *Charlson Comorbidity Index score was adjusted for multivariable logistic regressions. Values demonstrating statistical significance are bolded*.

**Table 3:** Z code use over time: Before and during COVID-19 (N = 1,190,531).

	**Before COVID-19**		**During COVID-19**		**Odds ratio/OR (95% CI)^a^**
**Characteristic**	**Z code use**	**No Z code use**	**Z code use**	**No Z code use**	
**Age group (years)**					
<18	1,152 (0.78)	147,343 (99.22)	1,361 (0.92)	147,134 (99.08)	**1.183 (1.093,1.280)**
18-29	1,398 (0.79)	176,172 (99.21)	1,514 (0.85)	176,056 (99.15)	**1.084 (1.007,1.166)**
30-39	1,270 (0.84)	150,446 (99.16)	1,453 (0.96)	150,263 (99.04)	**1.145 (1.062,1.235)**
40-49	1,152 (0.82)	140,015 (99.18)	1,259 (0.89)	139,908 (99.11)	**1.094 (1.009,1.185)**
50-59	1,271 (0.71)	178,045 (99.29)	1,399 (0.78)	177,917 (99.22)	**1.101 (1.021,1.189)**
60+	1,510 (0.38)	390,757 (99.62)	1,585 (0.40)	390,682 (99.60)	1.050 (0.978,1.127)
**Gender^b^**					
Female	3,602 (0.55)	651,804 (99.45)	3,899 (0.59)	651,507 (99.41)	**1.083 (1.035,1.133)**
Male	3,827 (0.81)	470,714 (99.19)	4,387 (0.92)	470,154 (99.08)	**1.148 (1.099,1.199)**
**Race^b^**					
White	3,252 (0.69)	468,546 (99.31)	3,807 (0.81)	467,991 (99.19)	**1.172 (1.118,1.228)**
Black	1,950 (0.76)	254,744 (99.24)	2,127 (0.83)	254,567 (99.17)	**1.092 (1.026,1.161)**
**Ethnicity^b^**					
Not Hispanic or Latino	3,616 (0.67)	540,079 (99.33)	4,042 (0.74)	539,653 (99.26)	**1.119 (1.069,1.170)**
Hispanic or Latino	160 (0.54)	29,683 (99.46)	175 (0.59)	29,668 (99.41)	1.094 (0.883,1.357)
**Residence^b^**					
Rural	924 (0.53)	172,522 (99.47)	985 (0.57)	172,461 (99.43)	1.066 (0.975,1.167)
Urban	6,749 (0.67)	995,649 (99.33)	7,423 (0.74)	994,975 (99.26)	**1.101 (1.065,1.138)**

a *The OR is estimated from logistic regression. It is the odds of Z code use before and during the pandemic within each demographic characteristic category (e.g., age group <18 years, age group 18-29 years, female subgroup, etc.)*.

b *Statistics of missing/unknown category in each variable and variables with cell size less than 20 are not reported. Values demonstrating statistical significance are bolded*.

### Z Codes by Healthcare Facilities

Patient data were from a total of 203 healthcare facilities, including 88 hospitals. Of the 203 facilities, SDoH were coded in 78 (38.4%) of them. Compared with hospitals that did not use Z codes (13; 14.8%), those that did use Z codes were more likely to have higher capacity as measured by number of beds (*p* = 0.017) and were also more likely to be teaching hospitals (*p* = 0.03), although the differences were significant only among the COVID-19 positive individuals (data not shown in Tables). There were only slight differences of the variable distribution when stratified by COVID-19 status. However, no significant associations were observed of the hospital characteristics (e.g., hospital bed size and teaching/non-teaching status) and Z-code use among the overall sample and COVID-19 negative population.

### Z Codes by Type of Patient Visit and Medical Specialty

As shown in Table 4, individuals who had inpatient visits (IP) were most likely to receive Z codes (OR: 5.26; 95%CI: 5.14–5.38; *p* < 0.0001); those at outpatient visits (OP) (OR: 0.07; 95%CI: 0.06–0.07; *p* < 0.0001) or emergency room visits (ER) (OR: 0.91; 95%CI: 0.89–0.93; *p* < 0.0001) were less likely to receive Z codes. Patients who visited emergency medicine (EM) (OR: 1.22; 95%CI: 1.19–1.25; *p* < 0.0001) and internal medicine providers (OR: 1.29; 95%CI: 1.24–1.34; *p* < 0.0001) had significantly higher odds of receiving Z codes.

**Table 4:** Distribution of Z codes utilization by type of patient visit and medical specialty.

**Items**	Overall number of Visits (N = 3,859,615^*^)	Visits without Z code (N = 3,831,027^*^)	Visits with Z code (N = 28,588^*^)	**OR (95%CI)**	***P-value***
**Patient visit type**					
**Emergency room visit**				**0.91 (0.89, 0.93)**	**<.0001**
No	1,796,904 (46.56)	1,782,937 (46.54)	13,967 (48.86)		
Yes	2,062,711 (53.44)	2,048,090 (53.46)	14,621 (51.14)		
**Inpatient visit**				**5.26 (5.14, 5.38)**	**<.0001**
No	3,287,220 (85.17)	3,272,161 (85.41)	15,059 (52.68)		
Yes	572,395 (14.83)	558,866 (14.59)	13,529 (47.32)		
**Outpatient visit**				**0.07 (0.06, 0.07)**	**<.0001**
No	2,954,909 (76.56)	2,926,914 (76.4)	27,995 (97.93)		
Yes	904,706 (23.44)	904,113 (23.6)	593 (2.07)		
**Top 9 medical specialty visits**				
**Emergency medicine**				**1.22 (1.19, 1.25)**	**<.0001**
No	2,643,531 (68.49)	2,625,213 (68.53)	18,318 (64.08)		
Yes	1,216,084 (31.51)	1,205,814 (31.47)	10,270 (35.92)		
**Internal medicine**				**1.29 (1.24, 1.34)**	**<.0001**
No	3,590,326 (93.02)	3,564,251 (93.04)	26,075 (91.21)		
Yes	269,289 (6.98)	266,776 (6.96)	2,513 (8.79)		
**Family practice**				**0.50 (0.47, 0.54)**	**<.0001**
No	3,620,276 (93.8)	3,592,611 (93.78)	27,665 (96.77)		
Yes	239,339 (6.2)	238,416 (6.22)	923 (3.23)		
**Gastroenterology**				**0.05 (0.04, 0.07)**	**<.0001**
No	3,649,809 (94.56)	3,621,310 (94.53)	28,499 (99.69)		
Yes	209,806 (5.44)	209,717 (5.47)	89 (0.31)		
**General surgery**				**0.26 (0.23, 0.29)**	**<.0001**
No	3,741,008 (96.93)	3,712,654 (96.91)	28,354 (99.18)		
Yes	118,607 (3.07)	118,373 (3.09)	234 (0.82)		
**Orthopedic surgery**				**0.14 (0.12, 0.16)**	**<.0001**
No	3,741,312 (96.93)	3,712,849 (96.92)	28,463 (99.56)		
Yes	118,303 (3.07)	118,178 (3.08)	125 (0.44)		
**Obstetrics & gynecology**				**0.26 (0.23, 0.30)**	**<.0001**
No	3,757,388 (97.35)	3,729,003 (97.34)	28,385 (99.29)		
Yes	102,227 (2.65)	102,024 (2.66)	203 (0.71)		
**Ophthalmology**				**0.004 (0.001, 0.014)**	**<.0001**
No	3,784,177 (98.05)	–	–		
Yes	75,438 (1.95)	–	–		
**Cardiovascular disease**				**0.45 (0.39, 0.51)**	**<.0001**
No	3,787,445 (98.13)	3,759,100 (98.12)	28,345 (99.15)		
Yes	72,170 (1.87)	71,927 (1.88)	243 (0.85)		

*Note: Per SC DHEC data use policy, categories with cell size less than 10 are not reported to protect patient privacy*.

* *The numbers in this table are the counts of clinical encounters not the unique patient counts. Values demonstrating statistical significance are bolded*.

**Figure 1:**
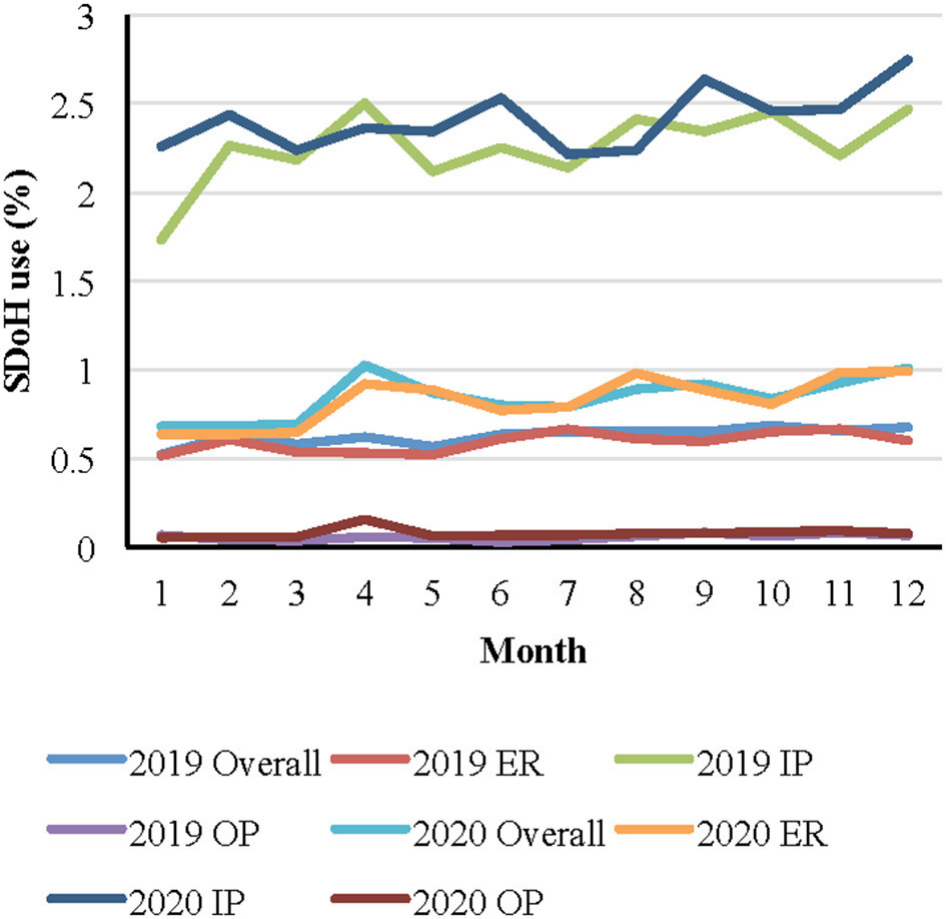
Monthly Z code use by visit type over time^*^ . ^*^Note: ER = emergency room visit; IP = inpatient visit; OP = outpatient visit.

**Figure 2:**
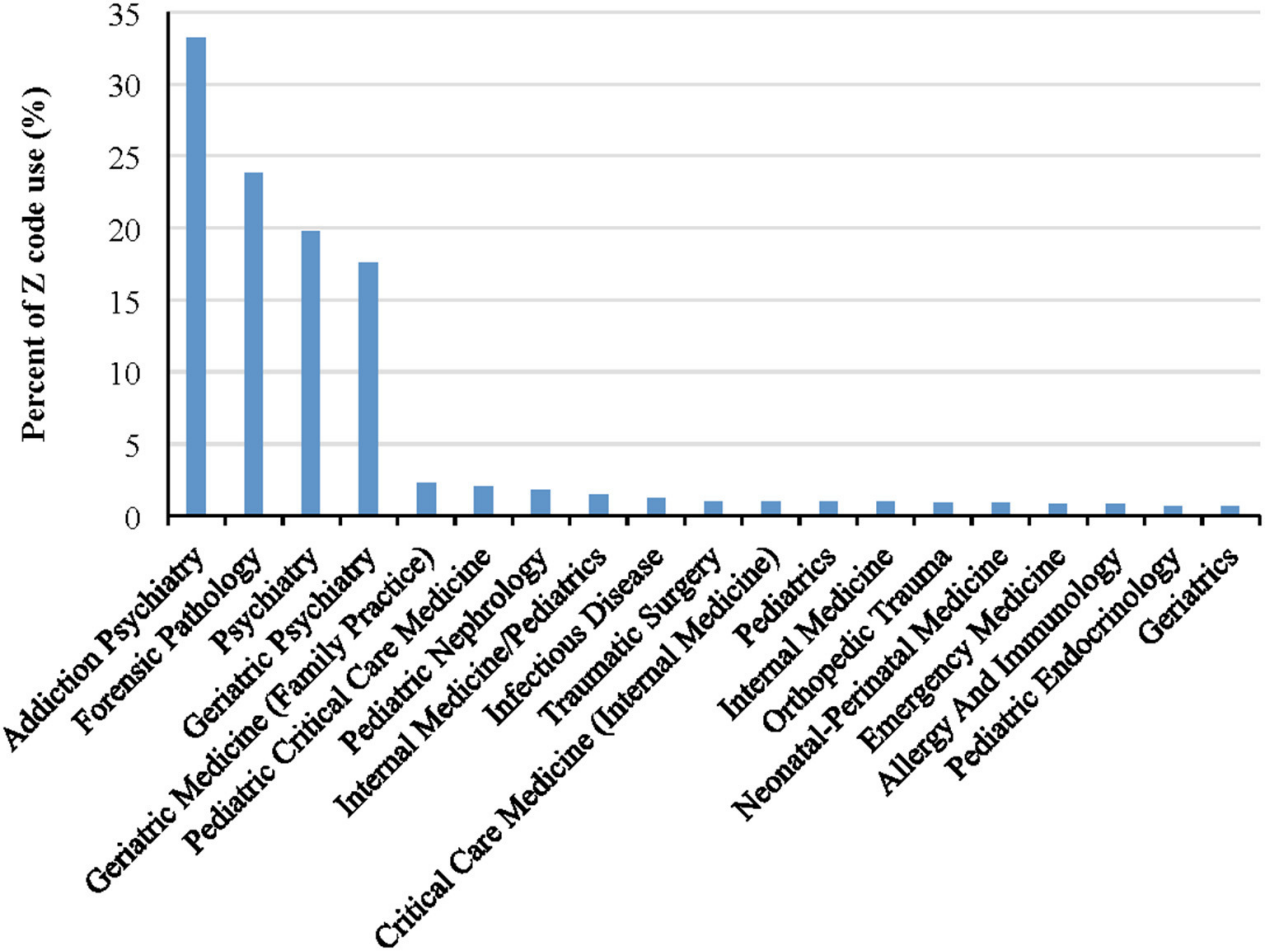
Percentage of clinical visits with Z code use by medical specialty.

As shown in Figure 1, there was a slight increase of Z code use from 2019 to 2020 (OR: 1.33, 95% CI: 1.30, 1.36; *p* < 0.0001), and the increase in Z code utilization was still significant when stratified by healthcare facility type across time (all *p*-values < 0.01). Figure 2 presents specialty clinic visits using Z codes most often, including addiction psychiatry/AP (33.2%), forensic pathology/FP (23.8%), general psychiatry (19.8%), and geriatric psychiatry (17.6%).

### Z Codes According to Type of Patient Visit, Specialty Type, and Racial and Ethnic Group

**Figure 3:**
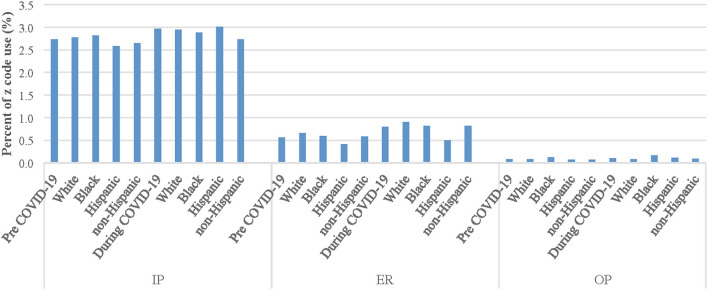
Z code use among racial and ethnic groups before and during COVID-19 by visit type. Note: ER = emergency room visit; IP = inpatient visit; OP = outpatient visit.

**Figure 4:**
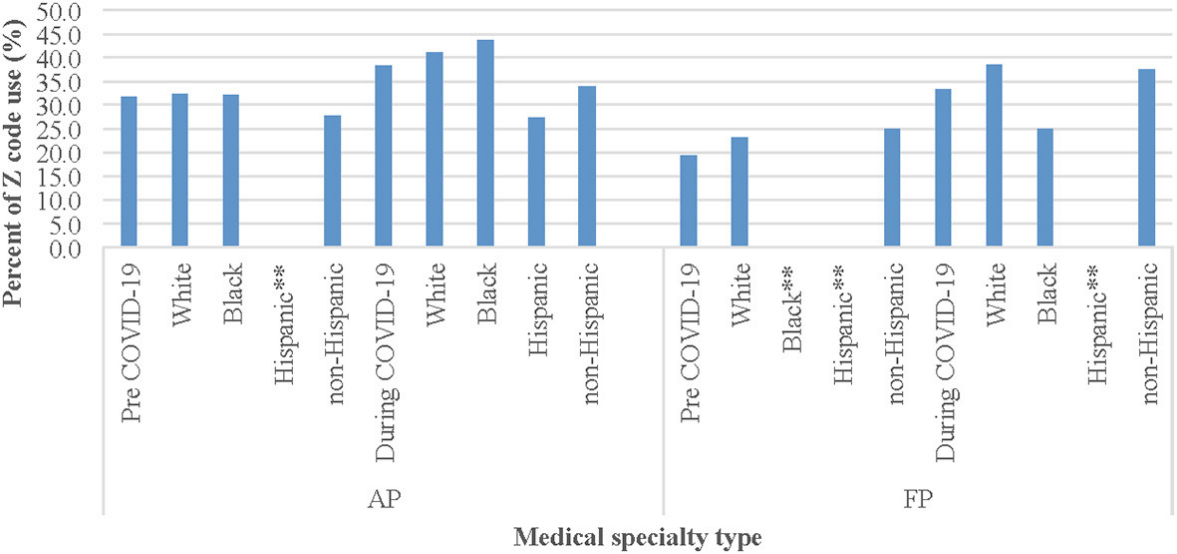
Z code use among racial and ethnic groups by medical specialty type before and during COVID-19^*^ . ^*^Note: Before COVID-19 period: 1/2/2019-3/5/2020; During COVID-19 period: 3/6/2020-1/14/2021. AP = Addiction Psychiatry; FP = Forensic Pathology. ^* *^Percentages of Z code use among these racial and ethnic groups in AP visit and FP visit were not available because there were no such visits/observations (as denominator).

Figures 3, 4 present Z coding by racial and ethnic group before and during COVID-19 according to visit type (IP, ER, and OP) and selected specialty types (AP and FP) among the full patient sample (data were similar when comparing COVID-19 positive and COVID-19 negative individuals). While there were no significant differences before the pandemic in coding between Black and White patients (OR: 1.01; 95%CI: 0.94, 1.09; *p* = 0.70), there was a slightly higher use of codes for IP visits among Black patients than White patients (2.82 vs. 2.78%). However, during the pandemic, there was a lower use of codes among Black patients than White patients (2.90 vs. 2.95%) for IP visits. Similarly, there were also no significant differences before the pandemic in coding between Hispanic and non-Hispanic patients for IP visits, although there was a lower use of codes among Hispanic patients than non-Hispanic (2.58 vs. 2.64%) and vice versa during the pandemic (3.00 vs. 2.74%) for IP visits.

Regarding the top two medical specialties (AP and FP) with the most patients who received any Z codes over time, the proportion of White patients receiving any Z codes increased significantly from 32.4% pre-COVID to 41.2% use during COVID (OR: 1.46; 95%CI: 1.07, 2.01; *p* = 0.02) and among Black patients (32.2 vs. 43.8%; OR: 1.64; 95%CI: 1.01, 2.64; *p* = 0.04) for AP. Because there were no visit observations in AP or FP clinics for Hispanic patients before the pandemic, comparison of Z code use before and during COVID-19 was unavailable for this ethnic group.

## Discussion

This study involved a population-level analysis of Z code use in one southeastern state before and during the COVID-19 pandemic. Findings clearly indicate ICD-10-CM codes focused on SDoH are underutilized in this large patient population. Our findings support the statement from the American Hospital Association: “*Despite the availability and utility of these ICD-10-CM codes, hospitals have not widely adopted the use of Z codes. Adoption has been limited due to a lack of clarity on who can document a patient's social needs, absence of operational processes for documenting and coding social needs, and unfamiliarity with Z codes. In addition, coders may need encouragement and support from hospital leaders to collect these codes that were once perceived as a lower priority”* (19).

Providers applied Z codes to only 1.23% of the entire study population. In comparison, a national survey of Z Code usage in 2017 indicated 2.4% of the sample received at least one code (24), and another comparing usage for Medicare fee-for-service beneficiaries between 2016 and 2019 found 1.31–1.59% of patients received Z codes (25). This suggests adoption of Z codes among South Carolina clinicians falls below national utilization levels (24) but is more comparable with certain studies (25). While not significant, certain Z code categories were applied slightly more among COVID-negative than COVID-positive patients.

Overall, the top Z code categories used involved housing and economic circumstances, problems related to upbringing, and issues related to primary support networks, including one's family situation, all salient contributors to poor health and impaired disease management. These codes, however, were still used rarely in our dataset of over 1.1 million individuals. Codes focused on education and literacy were also rarely utilized. Health literacy is a great predictor of healthcare outcomes compared with other demographic factors and is found to mediate other SDoH (26). The more individuals understand about their medical situation and steps they can take to actively engage in their health plan, the greater opportunity they will have for improved outcomes. The World Health Organization asserts that being well-informed about COVID-19 is critical for preventing and slowing transmission of the virus (27). This is of increased importance to combat mis- and disinformation and debunk community-wide misperceptions that contribute to economic and racial and ethnic health disparities. In addition, issues of mental health, and specifically depressive symptoms and depression, have increased significantly among racial and ethnic populations since the start of the pandemic (10, 28, 29). Additional studies are required to understand the potentially long-lasting health effects related to SDoH among underserved populations.

The greatest use of Z codes was found among mental health focused practices and IP services. This is not surprising given the association of several SDoH with mental health outcomes (4, 28, 29), IP service use (30), and the reciprocal nature of mental illness and disease severity on SDoH (31). In addition, more time is spent with patients during IP care, and there is greater likelihood of integration with social work and extensive discharge planning. There was an increasing trend of Z code use during the pandemic compared to before the pandemic both overall and stratified by patient visit type (e.g., ER, OP). It is interesting that patients visiting EM specialty were more likely to receive Z codes, while patients visiting the ER were less likely to receive Z codes. A potential explanation of such contradictory results is that not all ER visit services are provided by EM providers (22.76% of ED visits are served by other specialty providers) which may impact likelihood of Z code use. In addition, some EM providers provide other services than ER visits (only 30% of EM provider visits are from ER visits), and these non-ER visits may result in increased likelihood of Z code utilization. Additional research is needed to confirm this speculation.

The significantly lower odds of overall Z code use for individuals identified as Black or Hispanic is contradictory to the literature, as SDoH are the result of structural distribution of power, money, and resources, and heavily influenced by racial and ethnic inequality (32, 33). However, among all racial and ethnic groups, use of Z codes somewhat increased over time, with large significant increases for Black and White patients in AP settings. Individuals of all races and ethnicities experienced a variety of changes and losses due to COVID-19 restrictions and impacts on the national economy, to the detriment of their health and mental health, thus it makes sense that Z code application increased during COVID-19. In future research, it will be important to better understand trends for Hispanic individuals in AP settings, as research suggests much greater prevalence of depression, suicide ideation, and substance use for Hispanic individuals vs. Black, White, and other non-Hispanic individuals during the pandemic (28).

This study is one of the first to examine use of SDoH coding among a large patient population before and during the COVID-19 pandemic; however, there are some limitations. First, the data are from one large statewide cohort of individuals who were tested for COVID-19 in a southeastern state and cannot be generalized to other systems or locations. Second, we selected specific time points for categorizing before COVID and during COVID based on available data. We believe these ranges are appropriate given the timeframe when COVID-19 testing began in the United States. Third, this study did not address whether providers who coded for SDoH referred patients to follow-up resources based on the coding category. A mixed-method inquiry with providers regarding their understanding of Z codes and an examination of clinic flow and processes for making coding possible is being planned for a separate study. Fourth, This study did not analyze any associations between Z-code use and COVID-19 clinical outcomes (e.g., hospitalization, mortality). We will conduct such studies for next steps to examine how the disparities in social determinants of health contribute to COVID-19 clinical outcomes.

While SDoH coding was more common for mental health focused practices, the use of codes was still quite limited overall and learning more about providers' knowledge of SDoH and perceptions of their patients' social needs is critical for improved health outcomes. In addition, since teaching facilities were proportionally more likely to code for SDoH, additional efforts are needed to reach non-teaching facilities across the state with education about Z codes. To ensure we are considering SDoH in clinical encounters with patients, there is great opportunity for providers to partner with community resources, including shelters and housing authorities, employment centers, adult literacy centers, etc. Addressing social and environmental factors can lend to more meaningful encounters with patients and foster more comprehensive assessment and treatment planning which could result in fewer repeat visits, and ultimately, improved health outcomes.

## Data Availability Statement

The data analyzed in this study is subject to the following licenses/restrictions: Limited access to the data for this study is only available with approval from all data owners (including state agencies and health systems). Requests to access these datasets should be directed to BO, OLATOSI@mailbox.sc.edu.

## Ethics Statement

The studies involving human participants were reviewed and approved by University of South Carolina Institutional Review Board and relevant South Carolina state agencies. Written informed consent for participation was not required for this study in accordance with the national legislation and the institutional requirements.

## Author Contributions

XY led data analysis and contributed to data interpretation and manuscript writing. BY developed the manuscript and contributed to data interpretation. SC supported data analysis and contributed to manuscript edits. JZ advised on data analysis and contributed to manuscript edits. BO was involved with data acquisition. SQ and BO contributed to manuscript edits. XL contributed to the data analysis, data interpretation, and manuscript edits. DF led the overall study and contributed significantly to the manuscript. All authors approved the final manuscript.

## Funding

The research reported in this publication was supported by the National Institute of Allergy and Infectious Diseases of the National Institutes of Health (Award Number R01AI127203-4S1) and by The Duke Endowment (Award Number 6816-SP). The content is solely the responsibility of the authors and does not necessarily represent the official views of the National Institutes of Health or The Duke Endowment.

## Conflict of Interest

The authors declare that the research was conducted in the absence of any commercial or financial relationships that could be construed as a potential conflict of interest.

## Publisher's Note

All claims expressed in this article are solely those of the authors and do not necessarily represent those of their affiliated organizations, or those of the publisher, the editors and the reviewers. Any product that may be evaluated in this article, or claim that may be made by its manufacturer, is not guaranteed or endorsed by the publisher.
